# Computed tomography referral guidelines adherence in Europe: insights from a seven-country audit

**DOI:** 10.1007/s00330-024-11083-x

**Published:** 2024-10-10

**Authors:** Clara Singer, Mor Saban, Osnat Luxenburg, Lucia Bergovoy Yellin, Monika Hierath, Jacob Sosna, Alexandra Karoussou-Schreiner, Boris Brkljačić

**Affiliations:** 1 The Gertner Institute for Epidemiology and Health Policy Research, Chaim Sheba Medical Center, Tel Hashomer, Ramat-Gan, Israel; 2 Nursing Department, School of Health Sciences, Faculty of Medical and Health Sciences, Tel Aviv University, Tel Aviv, Israel; 3 Medical Technology, Health Information and Research Directorate, Ministry of Health, Jerusalem, Israel; 4 European Society of Radiology, Vienna, Austria; 5 Department of Radiology, Hadassah Medical Center, Faculty of Medicine, Hebrew University of Jerusalem, Jerusalem, Israel; 6 Radiation Protection Department, Health Directorate, Ministry of Health, Luxembourg City, Luxembourg; 7 Department of Radiology, University Hospital Dubrava, School of Medicine, University of Zagreb, Zagreb, Croatia

**Keywords:** Computed tomography, Referral guidelines, Appropriateness, Quality improvement, Clinical decision support

## Abstract

**Background:**

Ensuring appropriate computed tomography (CT) utilization optimizes patient care while minimizing radiation exposure. Decision support tools show promise for standardizing appropriateness.

**Objectives:**

In the current study, we aimed to assess CT appropriateness rates using the European Society of Radiology (ESR) iGuide criteria across seven European countries. Additional objectives were to identify factors associated with appropriateness variability and examine recommended alternative exams.

**Methods:**

As part of the European Commission-funded EU-JUST-CT project, 6734 anonymized CT referrals were audited across 125 centers in Belgium, Denmark, Estonia, Finland, Greece, Hungary, and Slovenia. In each country, two blinded radiologists independently scored each exam’s appropriateness using the ESR iGuide and noted any recommended alternatives based on presented indications. Arbitration was used in case auditors disagreed. Associations between appropriateness rate and institution type, patient’s age and sex, inpatient/outpatient patient status, anatomical area, and referring physician’s specialty were statistically examined within each country.

**Results:**

The average appropriateness rate was 75%, ranging from 58% in Greece to 86% in Denmark. Higher rates were associated with public hospitals, inpatient settings, and referrals from specialists. Variability in appropriateness existed by country and anatomical area, patient age, and gender. Common alternative exam recommendations included magnetic resonance imaging, X-ray, and ultrasound.

**Conclusion:**

This multi-country evaluation found that even when using a standardized imaging guideline, significant variations in CT appropriateness persist, ranging from 58% to 86% across the participating countries. The study provided valuable insights into real-world utilization patterns and identified opportunities to optimize practices and reduce clinical and demographic disparities in CT use.

**Key Points:**

***Question*** *Largest multinational study (7 EU countries, 6734 CT referrals) assessed real-world CT appropriateness using ESR iGuide, enabling cross-system comparisons*.

***Findings*** *Significant variability in appropriateness rates across institution type, patient status, age, gender, exam area, and physician specialty, highlighted the opportunities to optimize practices based on local factors*.

***Clinical relevance*** *International collaboration on imaging guidelines and decision support can maximize CT benefits while optimizing radiation exposure; ongoing research is crucial for refining evidence-based guidelines globally*.

## Background

Ionizing radiation optimization aims to ensure the benefits of exposures outweigh potential risks [1]. Computed tomography (CT) exams contribute by far the largest portion of radiation exposure from medical sources [2]. Appropriate imaging referrals not only reduce population exposure levels to ionizing radiation but also optimize healthcare resources. Several studies show suboptimal justification in current practice, with local and national audits reporting up to 39% [3] of CT exams as inappropriate and even higher rates seen in previous studies [4–6]. Prior research has identified several major factors contributing to the suboptimal justification and inappropriate use of CT imaging. A common issue is a lack of awareness or familiarity with established referral guidelines among referring clinicians. Variations in local practices and the absence of standardized justification processes across healthcare settings have also been reported as drivers of inappropriate CT utilization [7].

The justification and optimization of medical imaging using ionizing radiation is critical for patient safety while delivering clinical benefits. There is growing recognition of the need to ensure each CT exam is appropriate based on clinical need [8]. Retrospective reviews found inappropriate CT use rates ranging from 10% to 39% when referrals were assessed against clinical guidelines [3, 9–11]. Overall utilization of CT continues to rise rapidly due to its diagnostic capabilities and technological advances. However, ensuring each exam is truly medically indicated based on presenting symptoms or diagnostic questions becomes more vital as CT use increases at a population level.

Decision support tools could help promote evidence-based referral patterns and standardize appropriateness reviews [12–14]. The European Society of Radiology (ESR) iGuide clinical decision support system (CDSS) was developed to facilitate clinical decision-making for imaging referrals based on appropriateness criteria. It was developed in cooperation with the American College of Radiology (ACR) and includes Europeanized ACR appropriateness criteria which are evidence-based guidelines developed through expert consensus panels, that provide ratings to assist physicians in selecting the most appropriate imaging exams for specific clinical presentations [15, 16]. However, research evaluating the clinical implementation and success of such decision support tools, as well as the real-world performance of the ESR iGuide in assisting diagnostic decision-making and optimizing medical resource utilization at national scales, has been limited to date [14, 17–21].

Advancing the use of decision support systems is directly aligned with the goal of the European coordinated action on improving justification of computed tomography (EU-JUST-CT) project to coordinate efforts improving CT justification in Europe. The EU-JUST-CT project was a three-year, European Commission-funded initiative that was launched in 2021 [1, 21] and was led by the ESR. The project aimed to improve CT justification practices across Europe through multi-country audits, data collection, development of guidance, and knowledge sharing to help optimize appropriate diagnostic imaging usage on a broader scale.

The EU-JUST-CT project team developed a common methodology and tools for carrying out coordinated national/regional audits of diagnostic CT exams in adults and pediatric patients. The methodology integrated lessons from the project’s literature review and guidance from European regulatory and professional organizations [1, 8]. It defined procedures for sampling exams, auditing justification outcomes, and determining appropriateness rates (AR) against predefined standards. Specifically, the audit methodologies used successfully in Northern Ireland [22] and Luxembourg [3] were adapted.

The current audit aimed to evaluate if the process of justification is followed in the audited centers. A two-step methodology was developed to assess justification, beginning with centers completing a survey evaluating their written justification procedures. Second, the appropriateness of exams from about 1000 sampled consecutive referrals was assessed. The percentage of appropriate exams indicates justification implementation. Standardizing these audits allows benchmarking practices across European healthcare systems.

The survey of authorities and radiology societies in 30 European countries found variability in CT justification practices across those countries. Less than half reported advance justification by a medical practitioner before CT scans, though radiologists mostly perform daily justification even if often shared. While referral guidelines were widely available, they were reportedly used daily in justification decisions by referrers/practitioners in only a minority of countries across Europe [1].

The current project focused on assessing how well CT scan referrals adhere to appropriateness criteria in the ESR iGuide, a well-established CDSS for ordering imaging exams.

## Methods

The EU-JUST-CT project was conducted between April 2021 and March 2024 in seven EU member states: Slovenia, Belgium, Denmark, Estonia, Finland, Greece, and Hungary. See Appendices 1 and 2 for more information on the sampling method. Appendix 1 presents the dates of CT referral data collection and the number of referrals collected in each country. Appendix 2 provides details on the participating centers in each country, including the level of participation (national/regional), the number of hospitals/centers, the public/private distribution, whether they performed adult, pediatric, or both types of procedures, and estimates of the annual and daily volume of CT exams performed.

### Data collection

The data collection involved auditors assigned by each country’s National Competent Authority in radiation protection [23]. In each country, four auditors were assigned to review approximately 1000 anonymized CT referrals for CT examinations that had already been performed. The referrals selected represented consecutive CT examinations performed at each participating center over 1–2 workdays in 2022.

The 1000 referrals were divided into two groups, with each group sending up to two radiologist auditors from the same country but from different regions compared to the region where the sampled referrals originated. The aim was for each auditor to assess around 500 referrals, with each referral being evaluated by two auditors.

For each referral data was obtained concerning: patient age and sex, specialty of the referrer, examination requested, clinical background/reason for the examination, examination proposed by the referral guidelines, conclusion on the appropriateness of the examination, if examination not appropriate which type of examination would have been more appropriate.

### ESR iGuide use

The imaging referral guidelines of the ESR embedded in the ESR iGuide were used as a standard for the audits (April 2021 edition). The guidelines, covering all diagnostic imaging modalities, and over 2300 clinical scenarios, were in English language. Each auditor underwent an introductory session on how to use the system and accessed the guidelines using the ESR iGuide portal, with a username and password. Each auditor session was automatically assigned an identifier.

ESR iGuide defines a score of 1–3 as ‘inappropriate’, 4–6 as ‘partially appropriate’, and 7–9 as ‘fully appropriate’. When no match was found by the ESR, a score of 0 was assigned, and the referred CT was classified as inappropriate.

### Procedure

Institutional review board (IRB) approval was obtained at each participating center. Based on the CT referrals, the assigned auditors retrospectively assessed the appropriateness of the examinations.

To evaluate the appropriateness, the auditors scored each referral on a 1 (not recommended) to 9 (highly recommended) scale, using the ESR iGuide clinical decision support tool as the standard of reference. The auditors also noted if they identified a more appropriate exam recommended in the ESR iGuide compared to the exam that was requested. The auditors recorded the following information for each referral in a pre-defined Excel spreadsheet: the clinical indication or reason for the exam as it was entered in the ESR iGuide tool, the CT exam that was requested on the referral, if a matching exam was found in the ESR iGuide then the name of the corresponding recommended exam, the appropriateness score for the requested exam based on the ESR iGuide recommendations, and if a more appropriate alternative exam was identified in the ESR iGuide, the score for that higher recommended exam. This data collection process resulted in four Excel spreadsheets per country, with each spreadsheet containing information from approximately 500 cases that were reviewed by a different auditor.

### Data analysis

Initially, a comprehensive data cleaning process was carried out that included data validation and quality assurance on the Excel files received from each country. This was done to identify and address any data issues prior to analysis. According to the data cleaning phase and analysis planning, the ESR project office created a consolidated data file for each country.

During the data processing, two types of arbitration were performed by radiologists. In case of disagreement between the two auditors regarding the appropriateness level of a CT exam based on the ESR iGuide, the opinion of one of the two radiologists leading the project was obtained to resolve the discrepancy. Arbitration was also performed in cases where only one auditor audited a given referral. The second type of arbitration was performed in case of disagreement between the two auditors regarding the explanatory variables in each country (for example age, gender, or referrer specialty).

Additionally, in cases where the auditors could not find the clinical indication in the ESR iGuide and thus could not obtain a score, they could use their own expert opinion. Therefore, there were three categories of scoring: ESR iGuide-based, arbitration (discrepancy between auditors), or by expert opinion (not in ESR iGuide).

A dataset was built for statistical analysis for each country separately, including the definition of dependent and explanatory variables. Explanatory variables included grouped categories such as anatomical area and referring physician specialty.

The primary outcome measure was the AR, defined as the percentage of CT examinations deemed appropriate according to the ESR iGuide criteria. Both dichotomous (0–6 not appropriate, 7–9 appropriate) and categorical (0–3 inappropriate, 4–6 partially appropriate, and 7–9 fully appropriate) variables were used in the statistical analyses.

The dichotomous scoring provided a simple binary determination of whether the CT examination was deemed appropriate or not appropriate. The 3-categorical scoring allowed for a more nuanced evaluation, differentiating within the not appropriate category (0–6) between exams that are partially appropriate (4–6), and totally inappropriate (0–3). Examination justification assessment was conducted based on factors such as medical institution type (private/public), patient age group (children/adult), gender, patient status (inpatient/outpatient), referring physician specialty, and anatomical area. Pearson’s chi-square tests or Fisher’s exact test were used to assess statistical associations.

#### Inter-observed variability among auditors

According to ESR iGUIDE, referrals given a score of 7–9 were considered fully appropriate, referrals given a score of 4–6 were considered partially appropriate and referrals given a score of 1–3 were considered inappropriate. Where auditors scored a referral differently, analysis was conducted as to whether their scores placed the referral in the same category (in which case the auditors were considered to be in full agreement), adjacent categories (in which case the auditors were considered to be in partial disagreement), or opposite categories (in which case the auditors were considered to be in significant disagreement). Inter-observed variability between the two auditors was assessed in each country as a proportion of partial and significant disagreement from a total of audited referrals. According to the methodology of this study, variability between the two auditors was resolved by arbitration.

All statistical analyses were performed separately for each country using SAS software (version 9.1.3) and SPSS software (version 28) [17, 19].

### Ethical consideration

Approval was obtained from the local research ethics committees or IRBs at each of the 125 participating imaging facilities across the seven countries. At each site, the IRB either approved the study or waived the requirement for informed consent due to the retrospective nature and de-identification of the data.

Strict procedures were instituted to de-identify all patient data prior to its extraction from institutional hospitals’ electronic medical records and imaging systems for analysis. Names, medical record numbers, dates of birth, and other direct identifiers were removed to ensure patient confidentiality. The auditors and the project team only had access to aggregated data and could not link results back to individual patient information.

## Results

After cleaning and handling the data, a total of 6734 audited patient referrals for CT scans across 125 centers in seven countries were included in the audits. There were approximately 1000 records per country in the initial sample except for Finland, which had only 744 records, and Greece with 909 records. In general, few records were excluded from the audits (3% on average, a maximum of 7% in Denmark, minimum of approximately 1% in Slovenia and Estonia) due to invalid/duplicate records.

Additionally, on average approximately 9.5% of the audited CT examinations did not receive a score of appropriateness, due to a lack of or insufficient clinical information provided in the referral. There was large variability in this rate between countries, ranging from 0.3% in Finland up to 22% in Greece and 27% in Slovenia. If the clinical indications and the reason for the examination are absent from the referral, then it is impossible for the radiologist to evaluate the appropriateness of the requested CT examination. In this case, the requested CT examination cannot be justified.

After excluding invalid data (such as duplicates) and referrals that could not be scored due to insufficient clinical information, a total of 5899 referrals were ultimately evaluated and scored, representing about 88% of the total audited referrals across the participating countries (Table 1).

**Table 1:** Overview of the sample in the study countries

Country	TOTAL	Belgium		Denmark		Estonia		Finland		Greece		Hungary		Slovenia	
	*N*	*N*	% of total	*N*	% of total	*N*	% of total	*N*	% of total	*N*	% of total	*N*	% of total	*N*	% of total
Number of audited referrals (total)	6734	1006	100	1012	100	1013	100	744	100	909	100	1026	100	1024	100
Of which removed from analysis (duplicates, invalid data)	202	22	2.2	71	7	10	1	22	3	49	5.4	19	1.9	9	0.9
Of which unscored referrals (no/insufficient clinical data)	633	10	1	19	1.9	57	5.6	2	0.3	193	21.3	86	8.4	266	26.9^a^
Of which scored	5899	974	96.8	922	91.1	946	93.4	720	96.8	667	73.4	921	89.8	749	73.1

^a^ In Slovenia, we could not differentiate between insufficient data and “clinical reasons not found in iGUIDE” due to the deficiency of data (both were unscored)

Inter-observed variability between the two auditors was assessed in each country. Partial disagreement goes from 8% in Slovenia and Estonia to 18% in Hungary. Significant disagreement goes from 3% in Estonia and Finland to 12% in Denmark and 14% in Greece. See Appendix 3 for a full analysis.

**Fig. 1:**
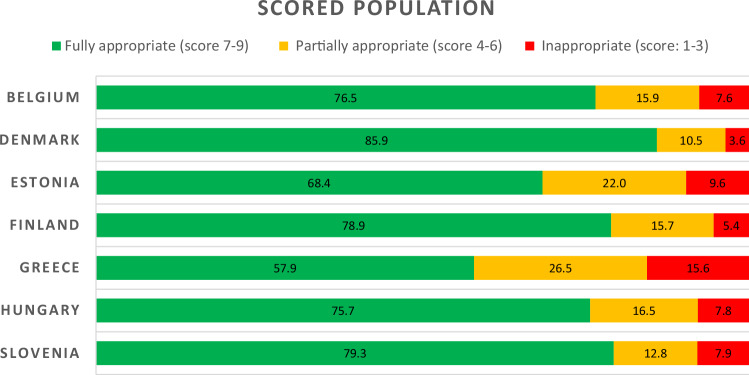
Percentage (%) of fully appropriate, partially appropriate, and inappropriate* CT examinations in each country among the scored population. * Inappropriate group includes cases that received a score of 0 due to a lack of match with the ESR iGUIDE exam recommendations

Of the examinations that were scored, the average proportion of fully appropriate CT examinations (score 7–9) was 75%, with the highest rate found in Denmark (86%), followed by Finland and Slovenia (~79%), Hungary and Belgium (~76%), Estonia (69%), and finally Greece with the lowest AR (58%). The average proportion of CT examinations found to be inappropriate (score 1–3) was 8.2% (minimum of 3.6% in Denmark and up to a maximum of 15.6% in Greece). Partially appropriate (score 4–6) rate ranges from 10.5% in Denmark to 26.5% in Greece (see Fig. 1).

Table 2 presents the association between explanatory variables and appropriateness level according to ESR as a binary variable, in each country according to the results of chi-square tests.

**Table 2:** Association between explanatory variables and AR according to ESR iGuide by country

	Belgium, (*N* = 974)		Denmark, (*N* = 922)		Estonia, (*N* = 946)		Finland, (*N* = 720)		Greece, (*N* = 667)		Hungary, (*N* = 921)		Slovenia, (*N* = 749)	
	Appr/total row	AR	Appr/total row	AR	Appr/total row	AR	Appr/total row	AR	Appr/total row	AR	Appr/total row	AR	Appr/total row	AR
Institution/*p* value	0.002^*,e^		0.62		0.0001^*^		0.18		< 0.001^*^				< 0.0001^*^	
Private	203/290	70	7/9	78	14/36	39	8/11	73	221/439	50	–		122/180	68
Public	542/684	79	785/913	86	633/910	70	560/709	79	165/228	72	697/921	76	472/569	83
Patient status/*p* value	0.06		0.15		0.0025^*^		–^b^		< 0.001^*^		0.0002^*^		0.05	
Inpatient/emergency	242/298	81	417/477	87	350/480	73	66/80	83	215/262	82	208/246	85	130/154	84
Outpatient	452/592	76	365/434	84	290/455	64	–		150/377	40	386/534	72	441/571	77
Undetermined^a^	51/84	61	10/11	91	7/11	64	526/667	79	21/28	75	103/141	73	23/24	96
Patient gender/*p* value	< 0.001^*^		0.06		0.025^*^		0.24		0.13		0.32		0.17	
Female	322/452	71	404/458	88	312/480	65	274/343	80	165/303	55	365/473	77	279/363	77
Male	421/518	81	386/460	84	333/464	72	291/373	78	206/341	60	327/440	74	287/354	81
Undetermined^a^	2/4	50	2/4	50	2/2	100	3/4	75	15/23	65	5/8	63	28/32	88
Patient age group/*p* value	0.03^*^		1		0.44		0.48		0.51		0.83		1	
Adult	729/947	77	788/917	86	641/939	68	555/706	79	384/665	58	692/913	76	561/712	79
Child	16/27	59	4/5	80	6/7	86	13/14	93	2/2	100	4/5	80	4/5	80
Undetermined^a^											1/3	33	29/32	91
Referrer specialty/*p* value	< 0.001^*^		0.0004^*^		< 0.0001^*^		–^c^		0.001^*^		< 0.0001^*^		0.0001^*^	
Family medicine	65/123	53	77/102	76	2/2	100	–		8/20	40	66/81	82	89/129	69
Brain related specialties	35/47	75	47/55	85	27/73	37	1/2	50	12/25	48	40/94	43	45/51	88
Emergency medicine	100/130	77	100/109	92	126/161	78	–		5/5	100	143/182	79	33/35	94
Internal medicine	275/339	81	187/219	85	62/89	70	1/2	50	127/242	53	150/199	75	124/146	85
Oncology	62/70	89	163/174	94	35/43	81	1/1	100	49/64	77	102/122	84	55/65	85
Radiology	–		–		–		–		–		–		48/72	67
Surgical specialties	169/219	77	207/248	84	89/123	72	3/4	75	71/119	60	146/185	79	118/144	82
Undetermined^a^	39/46	85	11/15	73	306/455	67	562/711	79	114/192	59	50/58	86	82/107	77
Referrer specialty (grouped)/*p* value	< 0.001^*^		0.001^*^		–^d^		–^d^		0.16		–^d^		0.0007^*^	
Family medicine	65/123	53	77/102	76	2/2	100	–		8/20	40	66/81	82	89/129	69
Specialist doctor	641/805	80	704/805	88	339/489	69	6/9	67	264/455	58	581/782	74	423/513	83
Undetermined^a^	39/46	85	11/15	73	306/455	67	562/711	79	114/192	59	50/58	86	82/107	77

* Statistically significant at the level of *p* ≤ 0.05

^a^ Undetermined or missing values may be due to missing data or inconsistencies between auditors in respect to that variable that could not be resolved by arbitration

^b^ No representation of outpatient status

^c^ No statistical test could be done for referrer specialty and ESR appropriateness due to very low frequencies of all cells (exact chi-square did not converge)

^d^ In Estonia, Finland, and Hungary family physicians cannot refer to CT, classification is not relevant for these countries

^e^ In Belgium, no hospital is 100% private or 100% public

### Belgium

Significant associations were found between the degree of appropriateness according to ESR iGuide and institution type (*p* = 0.002), gender of the patient (*p* < 0.001), age group of the patient (*p* = 0.03), and the expertise of the referring physician (*p* < 0.001). Higher AR was found in the public sector (79%) and for males (81%) compared to the private sector (70%) and females (71%). AR was much lower among children compared to adults (59% vs 77%, respectively), although only 27 children were included, and for general practitioners compared to specialists (53% vs 80%, respectively). A non-significant association was found between the degree of appropriateness according to ESR iGuide and the status of the patient, with higher AR for hospitalization compared to ambulatory care (81% vs 76%, respectively; *p* = 0.058).

### Denmark

A significant association was found between the degree of appropriateness according to the ESR iGuide and the specialty of the referring physician (*p* = 0.0004). Highest AR was observed for oncologists (93.7%) and emergency medicine doctors (91.7%). AR was lower for general practitioners as compared to specialists (75.5% vs 87.5%, respectively, *p* = 0.001).

### Estonia

A significant association was found between the degree of appropriateness according to ESR iGuide and belonging to a public or private institution (69.6% vs 38.9%, respectively, *p* = 0.0001), the status of the patient (*p* = 0.0018), gender of the patient (*p* = 0.0255), and the specialty of the referring physician (*p* < 0.0001), with a lower AR in the private sector and for ambulatory care compared to public sector and hospitalization (inpatient/emergency). Since there were only two general practitioners in the sample, we could not compare AR for general practitioners as compared to specialists. The reason for that is that in Estonia general practitioners cannot refer to CT. Appropriateness ratio was lower in female patients compared to male patients (65% vs 72%, respectively, *p* = 0.025). Highest AR was observed for oncology doctors (81.4%) and emergency medicine doctors (78.3%).

### Finland

For the data collected in Finland as part of this study, referrer information and inpatient/outpatient status were often lacking in the electronic medical records. No significant associations were found between the degree of appropriateness according to ESR iGuide and institution type, patient status, or gender. There were no ambulatory cases and 667 records (93%) were undetermined for patient status. No association was found for age group, but only 14 children were included in the study. No association was found between referrer specialty and ESR appropriateness. For referrer specialty, 99% of the records were undetermined. In Finland, general practitioners cannot refer to CT so a comparison between general practitioners and specialist doctors is not relevant.

### Greece

Significant associations were found between degree of appropriateness according to ESR iGuide and institution type, patient status, and referrer specialty (all three with *p* < 0.001). Higher ARs were found in the public sector (72%), in inpatient/emergency (82%) compared to private sector (50%), and in ambulatory care (40%). Referrer specialty was also found to be associated with AR, which was higher for oncologists (76.6%) and surgical specialties (60%) compared to internal medicine (52.5%), brain-related specialties (48%), and family medicine (40%). AR was much lower among adults compared to children (58% vs 100%, respectively; *p* = 0.34), though this was not significant, and since only two children were included in the study, no conclusion can be drawn regarding the association with AR. No associations with referrer specialty (grouped) were found for general physicians and for males compared to specialists (40% vs 58%, respectively; *p* = 0.16) and to females (55% vs 60%, respectively; *p* = 0.13), although there were only 20 referrals from general physicians in the sample.

### Hungary

A significant association was found between the degree of appropriateness according to ESR iGuide and the status of the patient (*p* = 0.0002) and the specialty of the referring physician (*p* < 0.0001). The highest AR was observed for oncologists (84%). In Hungary, general practitioners cannot refer to CT so a comparison between general practitioners and specialist doctors is not relevant and residents in emergency services referring patients were counted as general practitioners because they were not yet board-certified.

### Slovenia

Significant differences were demonstrated in ARs between private and public institutions (68% vs 83% respectively, *p* < 0.0001), between inpatient and ambulatory treatment (84% vs 77%, respectively, *p* = 0.05), and according to the referrer specialty (grouped) (83% for specialists vs 69% for general practitioners, *p* = 0.0007), as well as the referrer specialty (for example 94% among emergency medicine specialists vs 67% among radiologists, *p* = 0.0003).

**Fig. 2:**
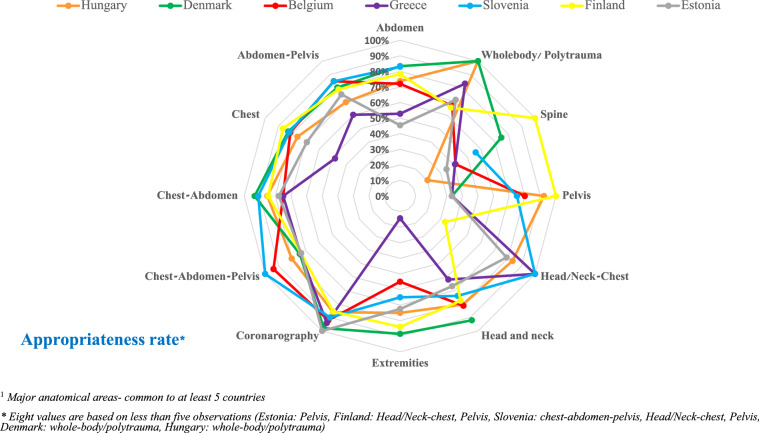
Radar chart showing AR for each anatomical area^1^ for CT requests by country

AR for each anatomical area for CT requests by country is shown in Fig. 2. The average AR in the ESR iGuide system by anatomical area was 76%. When examining the AR for exam types with more than five referrals analyzed, the highest AR was found for CT coronography (range of 86% to 100%). It should be noted that large variability in AR was found between countries and exam types. For example, spine CT (20% in Hungary up to 100% in Finland), pelvic CT (33% in Greece up to 92% in Hungary), and CT of the extremities (14% in Greece up to 88% in Denmark). In general, Greece and Estonia had relatively lower AR across all exam types compared to the other countries.

Table 3 presents the distribution of exam types where a more appropriate exam was recommended in each country. In 89% of cases of inappropriate CT examinations, on average, a different exam was recommended than the requested exam (83% in Estonia to 96% in Hungary). It was found that at least one auditor recommended one of four types of exams as a more suitable exam: MR (13–39%), X-ray (11–28%), other CT (7–26%), and US (3–10%). The highest percentage of exams recommended, instead of the referred CT, in each country were MR (35%, Belgium; 32%, Estonia; 39%, Slovenia; and 32%, Hungary), 2 + exams (35%, Denmark; 30%, Greece), X-ray (28%, Greece), other CT that the one referred (26%, Finland). It was found that performing at least two different types of exams was recommended in 7% (Estonia) to 35% (Denmark) of exams that the system recommended as more suitable than the requested exam. This situation may arise from disagreement between auditors.

**Table 3:** More appropriate exam distribution within each country where CT imaging was marked inappropriate (score less than 7), by exam type

Type of more appropriate exam	Belgium, (*N* = 229)		Denmark, (*N* = 130)		Estonia, (*N* = 299)		Finland, (*N* = 152)		Greece, (*N* = 291)		Hungary, (*N* = 224)		Slovenia, (*N* = 155)	
	*N*	% of total	*N*	% of total	*N*	% of total	*N*	% of total	*N*	% of total	*N*	% of total	*N*	% of total
No other exam was recommended	28	12.2	11	8.5	52	17.4	9	5.9	31	10.7	10	4.5	20	12.9
Another exam was recommended	201	87.8	119	91.5	247	82.6	143	94.1	250	85.9	90	95.5	135	87.1
MR	79	34.5	23	17.7	95	31.8	32	21.1	38	13.1	72	32.1	60	38.7
Other CT	16	7.0	22	16.9	51	17.1	40	26.3	23	7.9	37	16.5	27	17.4
X-ray	53	23.1	16	12.3	55	18.4	16	10.5	82	28.2	49	21.9	21	13.5
US	23	10.0	12	9.2	21	7.0	13	8.6	19	6.5	6	2.7	13	8.4
Other exam type^a^	1	0.4	1	0.8	3	1.0	1	0.7	1	0.3	2	0.9	0	0.0
2+ exams	29	12.7	45	34.6	22	7.4	41	27.0	87	29.9	48	21.4	14	9.0

^a^ i.e. NUC or PET-CT

## Discussion

This multinational large European project evaluated the appropriateness of CT referrals across seven different European healthcare systems using validated internationally known ESR iGuide criteria [24]. EU member states included in the project are from North, Central, East, and South Europe. Significant variations in AR were observed based on country, medical centers’ characteristics, patient demographics, exam type, and referring physician specialty [25].

Results of this audit show an average AR for CT of 75%, with the highest rate found in Denmark (86%) followed by Finland and Slovenia (~79%), Hungary and Belgium (~76%), Estonia (69%), and finally Greece with the lowest AR (58%). This is comparable with a 61% AR found in a smaller sample in Luxembourg for CT requests in a national audit on the appropriateness of CT and MRI examinations from 2019, and with a 63% AR found in Sweden published in 2023 [3, 20].

Higher appropriateness observed in public facilities aligns with prior research finding stricter guideline adherence where cost constraints are greatest [24, 26].

However, private centers may face pressures to approve CT exams to avoid losing patients which could influence referral decision-making.

The disparities observed between inpatient and outpatient ARs may be partially explained by differences in clinical documentation and oversight levels between these care settings [27, 28]. The lower AR observed for outpatient CT examinations compared to inpatient exams, though not statistically significant, suggests there may be room for improvement in referral practices for ambulatory care settings. Further research is needed to understand the factors contributing to potential differences in appropriateness between inpatient and outpatient referrals. Referral pathways present an opportunity for optimization, as initiatives targeting outpatient referral decision-making may help improve appropriateness. Recent advances in mobile technologies now facilitate new models for remote patient monitoring and coordinated exam planning across care settings [29, 30]. By providing clinicians with more comprehensive patient data and decision support tools, such technologies have the potential to enhance the appropriateness of imaging referrals, particularly in outpatient settings where access to prior clinical information may be more limited [31].

The surprising finding of lower appropriateness for female patients in some countries requires further exploration. Previous studies have reported potential overuse of medical imaging for women’s health, which may contribute to the lower ARs observed in this analysis [32]. However, the reasons underlying these gender-based differences warrant more in-depth investigation.

Future studies should incorporate more detailed patient-reported symptoms and clinical history to better understand the factors driving referral motivations across different patient demographics. Interestingly, the decreased CT use in European children mirrors declining referrals to CT in pediatric medicine, where MRI and ultrasound are used in preference, and the awareness of radiation risks to children is high among pediatric clinicians. In addition, the pediatric population in Europe is declining, and the older population increasing. Larger pediatric cohorts are needed to reliably gauge practices in this vulnerable population [33].

Appropriateness variability by exam type echoes prior reports attributing such heterogeneity to inconsistent criteria definitions and the auditors’ varying levels of familiarity with different types of CT examinations. Standardizing evaluation techniques and expanding virtual training modules could promote greater protocol harmonization Representing disease categories beyond cancers, such as investigating over/under age 50 subgroups, may broaden insights [18, 34].

Referral variations between specialties observed align with defensive medicine influencing referrals. Identified resource constraints divert referrals to accessible rather than optimal exams. This may conflict with the as low as reasonably achievable (ALARA) principle by overusing radiation-based exams [35].

As noted, evolving appropriateness guidelines like the ESR iGuide system used in this project need ongoing refinement to fully encompass real-world clinical variations. This is important to help further standardize reporting and guide more equitable, high-value imaging access according to need while still supporting radiation safety principles such as ALARA [1, 8, 35–37].

In this project, our main goal was to assess the justification for referring to a CT exam in general, without fully assessing compliance with requested body areas and imaging protocol details. For example, a referral for an abdominal–pelvic exam was considered justified even if ESR iGuide identified a chest–abdominal–pelvic exam as appropriate or vice versa. When auditors partially agreed on specified body areas, common regions or the broadest areas were chosen. Further research is needed to evaluate how aligning requested body areas and protocol affects the overall justification assessment [14, 17, 38, 39].

Our results highlight that on average a quarter of the CT examinations were not justified, and that radiation exposure of these patients was potentially avoidable. From a population perspective, further studies are needed to calculate the real percentage of misused radiation exposure based on study type as well as areas exposed. Further detailed studies are needed to evaluate the percentage of inappropriate CT examinations in Europe, based on the type of examination, area exposed and contrast media administration; such studies are relevant from the population and quality and safety from an imaging perspective.

Notable variability of appropriateness was associated with the region, healthcare setting, patient characteristics, and physician specialty—some aligning with prior reports, others offering a unique perspective given the comprehensive, international scope. Disparities between inpatient and outpatient settings, in particular, require targeted strategies to coordinate equitable care across the care continuum.

Dynamic living referral guidelines that account for real-world clinical diversity remain important for ensuring appropriateness. Larger datasets incorporating richer clinical indications through tools like natural language processing and large language models can strengthen risk stratification to better support complex decisions. Physician input on specialty- and system-specific factors ensures guidelines capture real-world practice.

At a policy level, monitoring appropriateness and utilization trends informs guideline refinement through data. Continued international research evaluating appropriateness methodology remains important to shaping practices and empowering sustainable healthcare through guidelines reflecting diverse practices. This innovative project paves the way forward.

### Limitations

There were several limitations to the audit design and implementation. Technical issues with the ESR iGuide system made it difficult for auditors to find matching clinical criteria, as some clinical indications were missing from the system such as for COVID-19 or the follow-up of specific oncological malignancies. A number of records had insufficient data, duplicates, or errors, significantly reducing the number available for analysis. Pediatric populations or non-specialist physicians were under-represented in the sample population challenging the comparisons between children and adults or between general practitioners of family medicine and specialists. Moreover, in three out of the seven countries (Estonia, Finland, and Hungary) general practitioners or family doctors cannot refer to CT. Comparisons between requested and ESR iGuide recommended exams were challenging. Contradictions in variable data (e.g., gender, age, hospitalized/ambulatory, and referred exam) necessitated blanking fields or arbitration.

Potentially, referrals do not include all clinical information and further evaluation of the entire medical record may be preferred. However, at this stage this task is challenging. The workflow process with the ESR iGuide could be improved, such as defining broader exam category choices for data entry. However, the large-scale multinational evaluation with meticulous sampling methodology may overcome those barriers partially.

## Conclusion

In conclusion, this first European multi-country assessment of CT appropriateness using the ESR iGuide criteria generated novel insights with implications for advancing evidence-based practice. The study identified important opportunities and disparities to guide the strategic optimization of CT utilization. There is a clear need for further improvement in the justification process internationally to reduce non-indicated radiation exposure.

## Supplementary information

ELECTRONIC SUPPLEMENTARY MATERIAL

## Abbreviations

AR: Appropriateness rate
CT: Computed tomography
ESR: European Society of Radiology
EU: European Union
EU-JUST-CT: European coordinated action on improving justification of computed tomography
IRB: Institutional Review Board
