# Supplementary material for: Computed tomography referral guidelines adherence in Europe: insights from a seven-country audit
Source: Eur Radiol. 2024 Oct 10;35(3):1166–77. doi: 10.1007/s00330-024-11083-x (PMC11835886; doi:10.1007/s00330-024-11083-x)
Supplement: Supplementary file 1 — ELECTRONIC SUPPLEMENTARY MATERIAL [file 330_2024_11083_MOESM1_ESM.pdf]

Appendix 1. Details on sampling

Country	Date(s) for collection	No. Referrals collected
Belgium	08/03/2022	1586
Denmark	07/02/2022 – 11/02/2022	1146
Estonia	Up to 20/04/2022	1154
Finland	30/03/2022	815
Greece	18/01/2022 – 19/01/2022	909
Hungary	31/01/2022 – 03/02/2022	1304
Slovenia	10/03/2022 – 11/03/2023	1723

Appendix 2. Details on Centers

Country	Level of Participation (country/region)	No. of participating hospitals (centres)	Of which public/ private	Of which adult/paediatric/both	CTs per country per year	Estimated CTs per day
**BE**	Representative selection of centres across the country	11	8 public,<br>3 private*	11 adult & paediatric	2,349,442	835 (Brussels region)
**DK**	Region of Southern Denmark	11	10 public, 1 private	10 adult, 1 adult & paediatric	1,057,470 adult<br>12,243 paed	1,000 (region)
**EE**	Entire country	19	17 public, 2 private	Mainly adult (only 1,200 paediatric CTs/per year in the entire country)	153,227 adult<br>1,287 paed	618 (country)
**FI**	Helsinki district	2 (17)	1 public,<br>1 private	16 both, 1 paediatric	570,000	892 (region)
**GR**	Thessaloniki region	26	8 public, 18 private	19 adult, 7 adult & paediatric,	1,500,000	950 (region)
**HU**	4 counties: Baranya, Csongrád, Hajdú-Bihar, Borsod-Abaúj-Zemplén	4 (21)	4 public,<br>0 private	adult & paediatric	1,500,000	500-1,000 (region)
**SI**	Entire country	20	16 public, 4 private	17 mostly adult, 3 adult & paediatric	165,000	660 (country)

Appendix 3: inter-observed variability among auditors in the study countries

**Country**	**TOTAL**	**Belgium**		**Denmark**		**Estonia**		**Finland**		**Greece**		**Hungary**		**Slovenia**	
	**N**	N	% of Total	N	% of Total	N	% of Total	N	% of Total	N	% of Total	N	% of Total	N	% of Total
**Number of audited referrals (Total)**	**6,734**	1,006	100	1,012	100	1,013	100	744	100	909	100	1,026	100	1,024	100
• Of which Full agreement	**4,398**	758	75.35	648	64.03	749	73.94	489	65.73	506	55.67	558	54.39	690	67.38
• Of which Partial Disagreement (Diff 1 Notch)	**767**	115	11.43	109	10.77	85	8.39	69	9.27	122	13.42	188	18.32	79	7.71
• Of which Significant Disagreement (Diff 2 Notch)	**493**	39	3.88	122	12.06	34	3.36	24	3.23	131	14.41	80	7.80	63	6.15
• Only one score (by one of the auditors)	**860**	72	7.16	62	6.13	135	13.33	140	18.82	101	11.11	181	17.64	169	16.5
• Other (invalid data/delete)	**216**	22	2.19	71	7.02	10	0.99	22	2.96	49	5.39	19	1.85	23*	2.24

**Includes 14 cases that one auditor did not audit and second auditor audited but gave no score*
